# Circ-RERE promotes autophagy and immune escape in acute myeloid leukemia involving the miR-128-3p/ZEB1/PD-L1 axis

**DOI:** 10.1016/j.clinsp.2025.100850

**Published:** 2026-03-20

**Authors:** XiaoWei Shi, TianTian Wang, KeYa Sha, ShuangYue Li, RenZhi Pei, Ying Lu

**Affiliations:** Department of Hematology, Yinzhou People's Hospital, Ningbo City, Zhejiang Province, China

**Keywords:** Circ-RERE, miR-128-3p, ZEB1, PD-L1, Acute myeloid leukemia, Autophagy

## Abstract

**Objective:**

To explore the role of circular RNA derived from RERE (circ-RERE) in regulating Programmed Death-Ligand-1 (PD-L1) expression and microRNA-128–3p (miR-128–3p)/Zinc finger e-box Binding homeobox-1 (ZEB1) axis in Acute Myeloid Leukemia (AML).

**Methods:**

AML cell viability, proliferation, apoptosis, the ratio of microtubule-associated protein 1A/1B-Light Chain 3-phosphatidylethanolamine conjugate (LC3-II) to free LC3 (LC3-I) (LC3-II/LC3-I), and PD-L1 expression, as well as CD8^+^*T*-cell cytotoxicity, were correspondingly analyzed. The targeting relationship between miR-128–3p and circ-RERE or ZEB1 was verified.

**Results:**

As detected, suppressing circ-RERE or overexpressing miR-128–3p significantly blocked the proliferation, migration, invasion, and autophagy of AML cells, promoted cell apoptosis, suppressed PD-L1 expression, and increased CD8+ *T*-cell cytotoxicity. circ-RERE competed with miR-128–3p to regulate ZEB1. Forced expression of ZEB1 did a reversal of circ-RERE suppression-induced effects.

**Conclusion:**

In a word, circ-RERE promotes autophagy and immune escape in AML by regulating PD-L1 expression through the miR-128–3p/ZEB1 axis, which may provide a new target for AML therapy.

## Highlights

• circ-RERE is augmented in AML.
• Silencing circ-RERE can inhibit autophagy and immune escape in AML cells.
• circ-RERE is a molecular sponge for miR-128–3p.
• miR-128–3p can block autophagy and immune escape in AML cells.
• Up-regulation of ZEB1 reverses the effect of down-regulation of circ-RERE on AML cells.

## Introduction

Acute Myeloid Leukemia (AML) is commonly accompanied by poor survival and poor prognosis. The etiology of AML is the abnormal changes of hematopoietic cells, which lead to the blocking of bone marrow differentiation and the inhibition of hematopoietic function.^1^ AML has marked genetic and epigenetic heterogeneity, as manifested by chromosomal translocations and hematopoiesis-associated gene recurrent mutations.^2^^,^^3^ Despite existing treatment options such as standard chemotherapy, outcomes remain poor, with 30 %–40 % of AML patients failing to respond to therapy or relapse.^4^^,^^5^ Recently, AML disease progression and relapse have been implicated in immune escape.^6^ Interestingly, studies provide unique insights into AML-specific immune escape mechanisms, many involving epigenetic changes.^6^^,^^7^

Noncoding transcripts have recently been a hotspot in research on the genetic and molecular mechanisms of cancer,^8^ and most of the human transcriptome is classified as noncoding RNAs (ncRNAs).^9^ As part of the endogenous ncRNA family, circular RNAs (circRNAs) have closed single-stranded and continuous loop structures.^10^ Some circRNAs have been found to function in AML, such as circ_100,053^11^ and circular RNA derived from RERE (circ-RERE).^12^ circ_RERE has been testified to be upregulated in multiple myeloma.^13^ Interestingly, the present research found an increase in circ_RERE expression in peripheral blood samples from AML patients, indicating its involvement in the progression of AML.

CircRNAs contain microRNA (miRNA) target sites, thereby participating in the pathogenesis of various diseases by interfering with miRNA.^13^^,^^14^ Furthermore, miRNAs are differentially expressed in biological processes and have diverse biological functions, including cell differentiation, apoptosis, and metabolism.^15^^,^^16^ circ-RERE has a binding site with microRNA-128–3p (miR-128–3p) through bioinformatics analysis based on the present research. Interestingly, miR-128–3p overexpression accelerates leukemia onset in a T-cell acute lymphoblastic leukemia mouse model.^17^ Also, Zinc finger e-box Binding homeobox-1 (ZEB1) has a binding site with miR-128–3p A previous report has found that ZEB1 is closely related to poor prognosis in AML patients.^18^ Based on the above findings, this study aimed to identify a novel circRNA/miRNA/mRNA network in AML and hypothesized that circ-RERE suppressed AML development through the miR-128–3p/ZEB1 axis.

## Methods

### Clinical sample collection

Peripheral blood from 120 newly diagnosed AML patients and 120 healthy donors without hematological tumors was collected in Yinzhou People's Hospital. With the written informed consent of patients, the study involving humans was approved by the Yinzhou People's Hospital (n° 201902CN16) research ethics committee. AML was diagnosed and classified according to the French, American, and British standards, as well as the World Health Organization criteria. STROBE guidelines were followed for the clinical part of this study.

### Cell culture

Cell culture, including human AML cell lines THP-1, Kasumi-1, and HL-60 and human normal bone marrow stromal cell line HS-5 (ATCC, USA) was done with 10 % FBS-RPMI-1640 medium (Gibco).

### Cell transfection

Small hairpin RNA targeting circ-RERE (sh-circ-RERE), overexpression circ-RERE, miR-128–3p mimic/inhibitor, along with the negative controls (sh-NC, oe-NC, mimic NC, and inhibitor NC) were all synthesized by GenePharma, while pcDNA-ZEB1 and negative control (pcDNA-NC) were by RiboBio. The transfection procedures performed on Kasumi-1 cells complied with the protocols of Lipofectamine 2000 (Invitrogen, USA).

### RNase R treatment

Total RNA was treated with RNase R (Seebio, China) or without RNase R (as a negative control) at 37 °C for 30 min. Thereafter, the treated RNA was subjected to reverse transcription quantitative polymerase chain reaction (RT‐qPCR) for the detection of circ-RERE and RERE expression.

### Actinomycin D assay

Kasumi-1 cells were cultured in a 24-well plate. Kasumi-1 cells were treated with 2.5 mg/L actinomycin D (Sigma‐Aldrich, USA). Cells were collected and RNA was extracted at 0 h, 4 h, 8 h, 12 h and 24 h. The stability and expression levels of circRNA and mRNA were examined by RT‐qPCR.

### Isolation and co-culture of CD8^+^*T*-cells

CD8^+^ *T*-cells were isolated from peripheral blood mononuclear cells of healthy volunteers using Dynabeads™ CD8 (Invitrogen) and tested by flow cytometry to determine the purity (> 90 %). After the addition of CD3 and CD28 monoclonal antibodies, CD8^+^ *T*-cells were co-cultured with Kasumi-1 cells in a two-chamber co-culture system (Millipore, USA), the upper chamber for Kasumi-1 cells and the lower one for CD8^+^ *T*-cells. RPMI-1640 medium supplemented with 10 % FBS (Gibco) was taken as the co-culture medium.

### RT-qPCR

Total RNA from blood and cells was isolated using FastKing One-Step RT-PCR Kit (Tiangen, China), treated with QuantiNova Reverse Transcription Kit (Qiagen, Germany) for reverse transcription, and tested by SYBR Green PCR Kit (Qiagen) for PCR. The 2^−ΔΔCT^ method was chosen to calculate gene mRNA expression. Table 1 presents primer sequences.

**Table 1:** PCR primer sequences.

Genes	PCR primer sequences (5′–3′)
circ-RERE	Forward: TATCGAGAGTCGGAGGCCAA
	Reverse: CACGAGGCAGTTAGCACTCA
miR–128–3p	Forward: GGTCACAGTGAACCGGTC
	Reverse: GCAGGGTCCGAGGTATTC
ZEB1	Forward: GCCAATAAGCAAACGATTCTG
	Reverse: TTTGGCTGGATCACTTTCAAG
U6	Forward: CTCGCTTCGGCAGCACA
	Reverse: AACGCTTCACGAATTTGCGT
GAPDH	Forward: CACCCACTCCTCCACCTTTG
	Reverse: CCACCACCCTGTTGCTGTAG

circ-RERE, circular RNA-RERE; miR-128–3p, microRNA-128–3p; ZEB1, Zinc finger E-Box-Binding homeobox-1; GAPDH, Glyceraldehyde-3-Phosphate Dehydrogenase.

### Western blot

Total proteins were obtained based on the radioimmunoprecipitation assay lysis reagent (Solarbio, China). After quantification with a bicinchoninic acid assay kit (Solarbio), the protein was separated on 10 % sodium dodecyl sulfate-polyacrylamide gel electrophoresis, transferred to polyvinylidene fluoride membranes for reaction with primary antibodies anti-ZEB1, anti-microtubule-associated protein 1A/1B-Light Chain-3 (LC3-I), anti-microtubule-associated protein 1A/1B-Light Chain-3-phosphatidylethanolamine conjugate (LC3-II), anti-Bcl-2-interacting protein-1 (Beclin-1), anti-sequestosome-1 (p62), anti-Programmed Death-Ligand-1 (PD-L1), and anti-Glyceraldehyde-3-Phosphate Dehydrogenase (GAPDH; 1:1000; Cell Signaling Technology, USA) and the goat anti-rabbit secondary antibody (Abcam, USA), and developed after treatment with enhanced chemiluminescence reagent (Beyotime, China).

### Methylthiazolyldiphenyl-tetrazolium bromide (MTT)

Kasumi-1 cells after 36 h of culture were supplemented with 20 μL of MTT (5 mg/mL; Sigma) for 4 h. Optical density value_570 nm_ was measured with a microplate reader (BioTek, USA) after the addition of dimethyl sulfoxide (Beyotime) to dissolve formazan precipitates.

### Colony formation assay

A 6-well plate was covered with Kasumi-1 cells (200 cells/well) for colony formation for 12d Then, the colonies were allowed to stain with crystal violet after methanol fixation for cell counting under a microscope (Leica, Germany).

### Flow cytometry

Kasumi-1 cells were stained using the fluorescein isothiocyanate-Annexin V Apoptosis Detection Kit (BD Biosciences, USA) and analyzed by flow cytometry.

### Cytotoxicity analysis

After co-culture, CD8^+^T-cell cytotoxicity was detected using the Lactate Dehydrogenase Cytotoxicity Kit (Thermo Fisher Scientific, USA).

### Dual-luciferase reporter gene assay

Complementary sites between miR-128–3p and circ-RERE or ZEB1–3′UTR were predicted on the starbase and mutated using the Quickchange XL site-directed mutagenesis kit (Agilent Stratagene, USA). Then, the Wild-Type (WT) and Mutant (MUT) of circ-RERE and ZEB1–3′UTR were inserted into pGL4 Luc-Rluc (BioVector-NTCC, China) to form WT-circ-RERE, MUT-circ-RERE, WT-ZEB1–3′UTR, and MUT-ZEB1–3′UTR. The vectors, along with miR-128–3p mimic or mimic NC, were co-transfected into Kasumi-1 cells to determine luciferase activity by a dual-luciferase reporter gene detection system (Genomeditech, China).

### Chromatin immunoprecipitation (ChIP)-qPCR assay

The ChIP assay was carried out according to the instructions of the ChIP assay kit (Cell Signaling Technology). Briefly, 4 × 10^6^ cells were treated with 1 % formaldehyde to cross-link proteins to DNA, followed by nucleus preparation and chromatin digestion; digested cross-linked chromatin was immunoprecipitated overnight (anti-ZEB1, 1:50, Abcam; Rabbit immunoglobulin G, Cell Signaling Technology), then chromatin was eluted from the precipitate and uncross-linked; the DNA was then purified, and qPCR was performed to detect the enrichment of DNA.^19^

### Tumor xenograft assay

The animal experiments were approved by the Animal Ethics Committee and performed following the Guide for the Care and Use of Laboratory Animals. Female BALB/c immunocompetent mice (5-weeks-old) were commercially provided by Guangzhou Experiment Animal Center (China). The mice were housed at (22±1) °C with a 12-hour light/dark cycle. Food and water were provided *ad libitum*.

Kasumi-1 cells transfected with sh-NC or sh-circ-RERE were subcutaneously injected into the BALB/c immunocompetent mice. Tumor size was measured every 7 days, with volume calculated using the volume=(length×width2)/2. On day 28, mice were euthanized, and tumors were harvested. After dissection, tumors were weighed and stored at −80 °C.

### Immunohistochemistry (IHC)

CD8^+^T-cells in xenograft tumor tissues of mice were evaluated by IHC. In brief, xenograft tumor tissues were processed with fixation and paraffin embedding in sequence in accordance with the standard procedures. Thereafter, xenograft tumor tissues were prepared into 5 µm sections. After deparaffinization and rehydration, the sections were treated with 3 % H_2_O_2_ for 15 min and then with 5 % normal goat serum for 30 min. Subsequently, rabbit anti-CD8 primary antibody (1:100, ab85792, Abcam, China) was evenly dripped onto each section to probe for 12 h at 4 °C. Thereafter, the horseradish peroxidase-labeled antirabbit secondary antibody (1:200, ab6721, Abcam) was added to incubate the sections for 30 min. Later, 3,3-diaminobenzidinetetrahydrochloride was added for color development for 10 min. After dehydration and transparentizing, the sections were sealed in neutral resin. At last, CD8^+^T-cells were presented as brown particles under the microscope.

### Statistical analysis

Data were obtained from at least three independent experiments and processed by GraphPad Prism 10. Categorical variables were analyzed by the Chi-Squared test and/or Fisher’s exact tests. The normality of continuous variables was evaluated by the Shapiro-Wilk test. Non-normally distributed continuous variables were analyzed with the Mann-Whitney *U* test, while normally distributed continuous variables were examined by Student's *t*-test or one-way analysis of variance. A *p* < 0.05 was defined as statistically significant.

## Results

### circ-RERE is augmented in AML

**Fig. 1:**
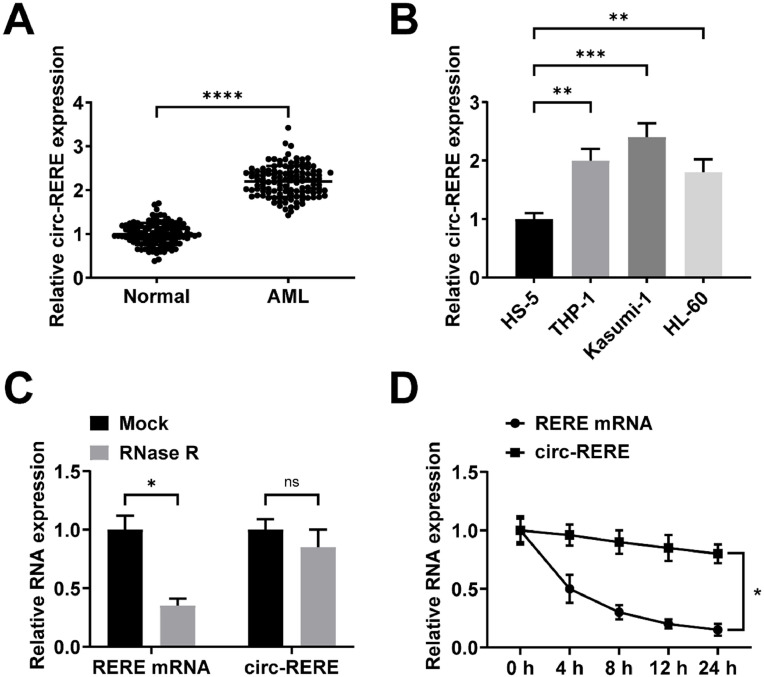
**circ-RERE is up-regulated in the peripheral blood of AML patients and AML cell lines.** RT-qPCR to detect circ-RERE in peripheral blood of AML patients (A), AML cell lines and normal bone marrow stromal cells (B). Data are presented as mean ± standard deviation (*n* = 3). * *p* < 0.05; ** *p* < 001; *** p 0.001; **** *p* < 0.0001.

RT-qPCR found that circ-RERE was up-regulated in the peripheral blood of AML patients (Fig. 1A). Based on the median expression level of circ-RERE, AML patients were divided into high and low circ-RERE expression groups. Statistical analysis demonstrated that circ-RERE expression correlated with platelet counts and FAB classification (Table 2). Furthermore, elevated circ-RERE expression was observed in AML cell lines (THP-1, Kasumi-1, and HL-60) (Fig. 1B). Since Kasumi-1 cells showed the most pronounced upregulation of circ-RERE (Fig. 1B), they were selected for subsequent transfection experiments. The circular nature and stability of circ-RERE were confirmed through RNase R and actinomycin D assays. circ-RERE exhibited greater resistance to RNase R digestion compared to its linear counterpart, verifying its circular structure (Fig. 1C). Actinomycin D treatment revealed that circ-RERE had a half-life exceeding 24 h, while the linear RNA degraded within 8 h (Fig. 1D). These results collectively demonstrate that circ-RERE exists in a stable circular form.

**Table 2:** Correlation of circ-RERE expression with clinical characteristics in AML patients.

Clinical factors	circ-RERE expression		p-value
	High (*n* = 60)	Low (*n* = 60)	
Gender			0.7086
Male	35	38	
Female	25	22	
Age, median (range), years	55 (16‒82)	60 (28‒85)	0.2830
WBC, median (range), × 10^9^/L	44.8 (0.4‒527.5)	54.5 (1.1‒208.0)	0.3598
Hemoglobin, median (range), g/L	76 (35‒142)	81 (40‒120)	0.6274
Platelets, median (range), × 10^9^/L	33 (5‒410)	54 (10‒384)	0.0207
BM blasts, median (range), %	45.9 (1.0‒96.0)	33.4 (5.0‒82.0)	0.0632
CR (±)	36/24	40/20	0.5701
FAB classification			0.0015
M0	0	0	
M1	0	3	
M2	16	29	
M3	5	10	
M4	23	13	
M5	16	5	
M6	0	0	
M7	0	0	
Cytogenetics			0.4454
Favorable	8	5	
Intermediate	36	43	
Poor	16	12	

WBC, White Blood Cell; BM blast, Bone Marrow Blast; CR, Complete Remission; FAB, French-American-British criteria.

### Silencing circ-RERE can inhibit autophagy and immune escape in AML cells

**Fig. 2:**
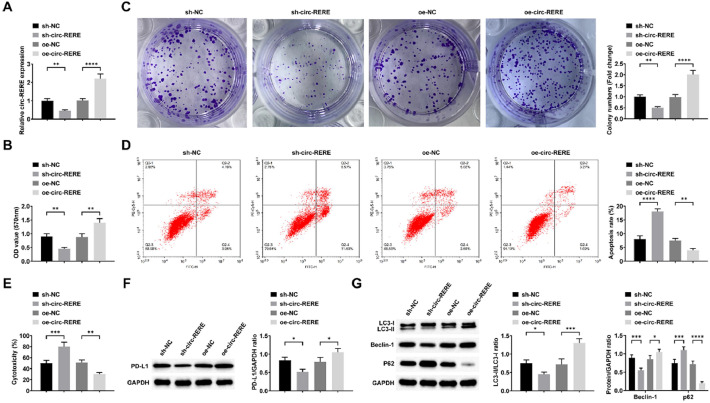
**Silencing circ-RERE can inhibit autophagy and immune escape in AML cells.** RT-qPCR to confirm circ-RERE expression change (A), followed by observations of viability (B), proliferation (C), apoptosis (D), cytotoxicity of CD8^+^*T*-cells (E), PD-L1 expression (F), LC3-II/LC3-I ratio and Beclin-1 and p62 expression (G). Data are expressed as mean ± standard deviation (*n* = 3). * *p* < 0.05, ** *p* < 0.01, *** *p* < 0.001, **** *p* < 0.0001.

To investigate the functional role of circ-RERE in AML, Kasumi-1 cells were transfected with either sh-circ-RERE or oe-circ-RERE, with RT-qPCR confirming successful transfection through corresponding decreases or increases in circ-RERE expression (Fig. 2A). Functional assays demonstrated that circ-RERE silencing significantly suppressed Kasumi-1 cell viability and proliferation (Fig. 2B‒C), while enhancing both cellular apoptosis and CD8+ *T*-cell-mediated cytotoxicity (Fig. 2D‒E). Western blot analysis revealed that circ-RERE deficiency reduced PD-L1 expression in Kasumi-1 cells (Fig. 2F). Furthermore, circ-RERE knockdown inhibited cellular autophagy, as evidenced by decreased LC3-II/LC3-I ratio and Beclin-1 expression coupled with increased p62 accumulation (Fig. 2G). Notably, all these effects were conversely observed upon circ-RERE overexpression (Fig. 2B-G), collectively indicating circ-RERE's critical role in regulating autophagy and immune evasion in AML cells.

### circ-RERE is a molecular sponge for miR-128–3p

**Fig. 3:**
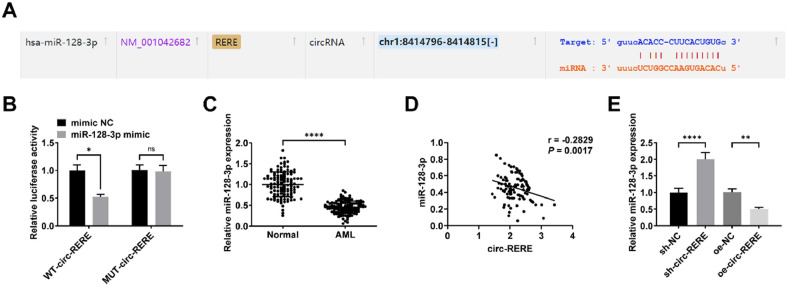
**circ-RERE is a molecular sponge for miR-128–3p** Predicted binding sites of circ-RERE and miR-128–3p on the starbase (A). Luciferase activity of WT/MUT-circ-RERE after miR-128–3p intervention (B). RT-qPCR detection of miR-128–3p expression in peripheral blood of AML patients (C); Correlation analysis of circ-RERE and miR-128–3p expression in AML peripheral blood (D); RT-qPCR detection of miR-128–3p expression after intervening circ-RERE expression (E). Data are expressed as mean ± standard deviation (*n* = 3). * *p* < 0.05, ** *p* < 0.01, *** *p* < 0.001, **** *p* < 0.0001.

Bioinformatic analysis using the starbase database predicted a binding site between circ-RERE and miR-128–3p (Fig. 3A). Luciferase reporter assays showed significantly decreased activity upon co-transfection of Wild-Type circ-RERE (WT-circ-RERE) with miR-128–3p mimics (Fig. 3B). Notably, miR-128–3p expression was downregulated in peripheral blood samples from AML patients and showed an inverse correlation with circ-RERE levels (Fig. 3C‒D). Furthermore, miR-128–3p expression was upregulated in Kasumi-1 cells transfected with sh-circ-RERE, while its expression was suppressed in oe-circ-RERE-transfected cells (Fig. 3E).

### miR-128–3p can block autophagy and immune escape in AML cells

**Fig. 4:**
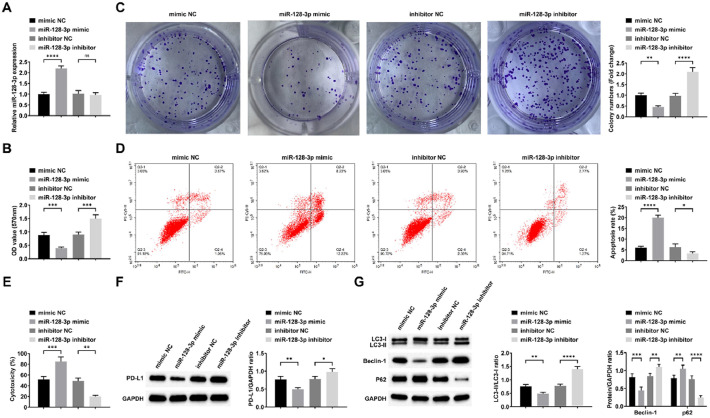
**miR-128–3p can inhibit autophagy and immune escape in AML cells.** RT-qPCR to confirm miR-128–3p expression change (A), followed by observations of viability (B), proliferation (C), apoptosis (D), cytotoxicity of CD8^+^*T*-cells (E), PD-L1 expression (F); LC3-II/LC3-I ratio and Beclin-1 and p62 expression (G). Data are expressed as mean ± standard deviation (*n* = 3). * *p* < 0.05, ** *p* < 0.01, *** *p* < 0.001, **** *p* < 0.0001.

To investigate miR-128–3p's function in AML cells, Kasumi-1 cells were transfected with either miR-128–3p mimic or inhibitor, with transfection efficiency verified by RT-qPCR (Fig. 4A). Functional assays demonstrated that miR-128–3p overexpression significantly suppressed cell viability and proliferation, while enhancing apoptosis and CD8+ *T*-cell-mediated cytotoxicity. Furthermore, it reduced PD-L1 expression and inhibited cellular autophagy (Fig. 4B‒G). Conversely, miR-128–3p knockdown produced opposite effects in Kasumi-1 cells (Fig. 4B‒G).

### miR-128–3p targets the regulation of ZEB1 expression

**Fig. 5:**
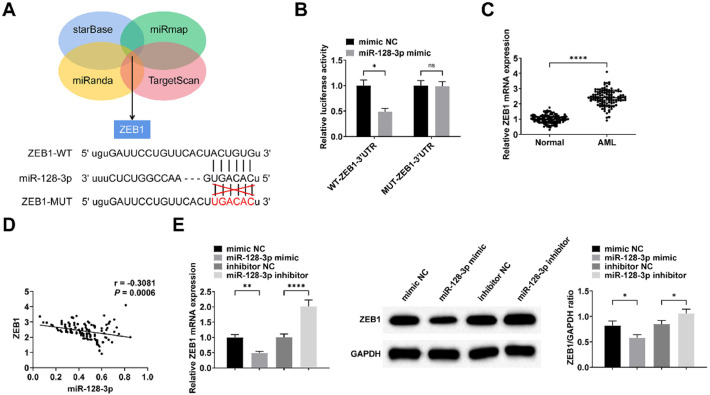
**miR-128–3p targets regulation of ZEB1 expression.** Predicted binding sites of ZEB1 and miR-128–3p (A). Luciferase activity of WT/MUT-ZEB1 after miR-128–3p intervention (B). RT-qPCR detection of ZEB1 expression in peripheral blood of AML patients (C); Inverse correlation between miR-128–3p and ZEB1 expression in AML patients (D); RT-qPCR and Western Blot detection of miR-128–3p expression after intervening miR-128–3p expression (E). Data are expressed as mean ± standard deviation (*n* = 3). * *p* < 0.05, ** *p* < 0.01, *** *p* < 0.001, **** *p* < 0.0001.

Bioinformatic analysis using multiple prediction tools (starBase, miRmap, miRanda, and TargetScan) identified ZEB1 as a potential downstream target of miR-128–3p (Fig. 5A). This targeting relationship was experimentally confirmed by dual-luciferase reporter assays (Fig. 5B). Consistent with these findings, ZEB1 expression was significantly upregulated in peripheral blood samples from AML patients and showed an inverse correlation with miR-128–3p levels (Fig. 5C‒D). Furthermore, transfection with miR-128–3p mimic or inhibitor resulted in corresponding downregulation or upregulation of ZEB1 expression in Kasumi-1 cells, respectively (Fig. 5E).

### Up-regulation of ZEB1 reverses the effect of down-regulation of circ-RERE on AML cells

**Fig. 6:**
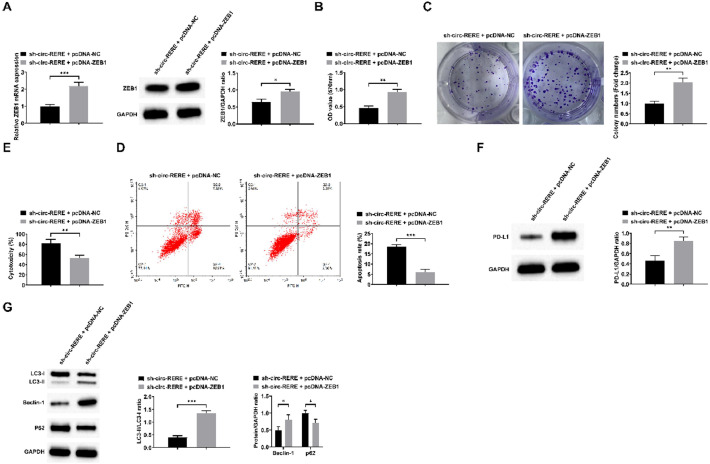
**ZEB1 involves circ-RERE-mediated regulation of autophagy and immune escape in AML cells.** RT-qPCR and Western Blot to confirm ZEB1 expression change (A), followed by observations of viability (B), proliferation (C), apoptosis (D), cytotoxicity of CD8^+^*T*-cells (E), PD-L1 expression (F); LC3-II/LC3-I ratio and Beclin-1 and p62 expression (G). Data are expressed as mean ± standard deviation (*n* = 3). * *p* < 0.05, ** *p* < 0.01, *** *p* < 0.001, **** *p* < 0.0001.

The functional role of the circ-RERE/miR-128–3p/ZEB1 axis was investigated through co-transfection of Kasumi-1 cells with sh-circ-RERE and pcDNA-ZEB1, with successful transfection confirmed by RT-qPCR and Western blot analysis (Fig. 6A). Functional assays demonstrated that pcDNA-ZEB1 effectively reversed the sh-circ-RERE-induced effects, including: (1) Decreased cell viability and proliferation, (2) Increased apoptosis and CD8+ *T*-cell cytotoxicity, and (3) Reduced PD-L1 expression and autophagy activity (Fig. 6B‒G). Furthermore, bioinformatic prediction using the JASPAR database identified multiple ZEB1 binding sites in the PD-L1 promoter region (Supplementary Fig. 1A‒B), which was experimentally validated by ChIP-qPCR showing direct ZEB1 binding to the PD-L1 promoter (Supplementary Fig. 1C).

### Circ-RERE knockdown suppresses tumor growth *in vivo*

**Fig. 7:**
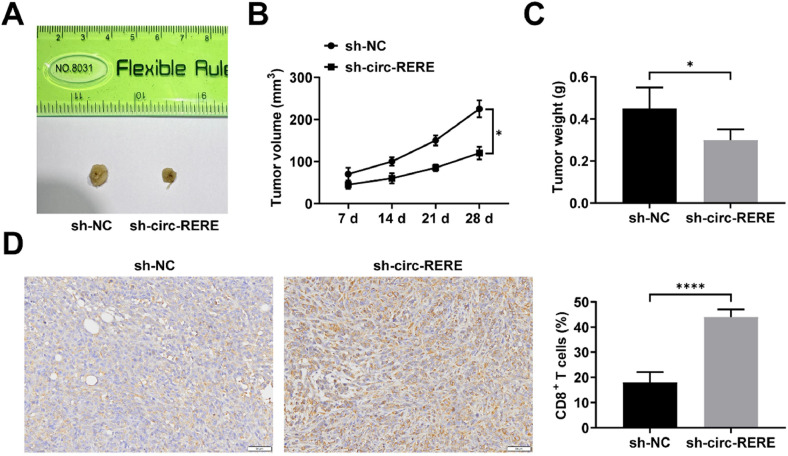
**circ-RERE knockdown suppresses tumor growth *in vivo*.** Representative images of xenograft tumors (A); Tumor volume measurements (B); Tumor weight analysis (C); CD8+ *T*-cell infiltration by immunohistochemical staining (D). Data are expressed as mean ± standard deviation (*n* = 5). * *p* < 0.05, ** *p* < 0.01, *** *p* < 0.001, **** *p* < 0.0001.

Kasumi-1 cells transfected with either sh-NC or sh-circ-RERE were subcutaneously injected into immunocompetent BALB/c mice. The results demonstrated that circ-RERE knockdown significantly reduced both tumor volume and weight (Fig. 7A‒C). Immunohistochemical analysis further revealed enhanced CD8+ *T*-cell infiltration in tumors with circ-RERE silencing (Fig. 7D).

## Discussion

CircRNAs are the focus of increasing research interest due to their characteristics^20^ and circRNA dysregulation in AML is functionally important and can influence disease onset and progression.^21^, ^22^, ^23^, ^24^ This study investigated and finally discovered that circ-RERE was up-regulated in peripheral blood samples of AML patients and AML cells. Importantly, circ-RERE exerts oncogenic effects by regulating post-transcriptional translation.

The tumor microenvironment allows tumor cells to infiltrate lymphatics and blood vessels, evade immunity, and resist to antigen-specific T cells, thereby achieving distant metastasis and growth.^25^ A key immune checkpoint receptor, PD-1, principally expresses on some activated cells, called T, B, Dendritic (DC), Natural Killer (NK), and Treg. PD-1 is also associated with increased Treg-cell proliferation and enhanced immunosuppressive function.^26^ After the recognition of tumor antigens by peripheral T-Cell Receptors (TCRs), PD-L1 will bind PD-1 and activate downstream-related signaling pathways, blocking TCR signaling via feedback inhibition, and downregulate the expression of specific antiapoptotic protein molecules and pro-inflammatory factors, ultimately inhibiting T-cell survival, proliferation, and immune function.^27^ The blockade of PD-1 and PD-L1 has been determined to be of great benefit in the immunotherapy of AML.^28^ The present study provided evidence that circ-RERE deficiency reduced PD-L1 expression in AML cells and stimulated CD8^+^ *T*-cell cytotoxicity, suggesting that circ-RERE-regulated immune escape is mediated by preventing CD8^+^ *T*-cells from normal function.

For rapid proliferation and turnover, cancer cells generate high autophagic flux. Recent reports suggest that reduced autophagy gene expression may stimulate AML proliferation^29^ and that autophagic flux is associated with time to disease remission in AML.^30^ Autophagy refers to a process of continuous self-renewal and active proliferation that is required for normal hematopoiesis.^31^^,^^32^ As echoed here, silencing circ-RERE could inhibit autophagy gene expression in AML cells to inhibit AML cell proliferation and promote apoptosis.

Mechanistically, it is generally believed that circRNAs can compete with miRNAs to affect the functions of miRNAs.^33^ Consistent with the theory, miR-128–3p was sponged by circ-RERE here. MiR-128–3p acts as an oncogenic miRNA,^34^ and can even regulate breast cancer progression.^35^ Notably, miR-128–3p hampered the viability, proliferation, and autophagy of AML cells, whereas it drove apoptosis. This study further verified that miR-128–3p represses immune escape in AML cells by blocking PD-L1.

ZEB1 was originally identified as a lens-specific binding protein for the δ1-crystal enhancer and is a key transcription factor during the epithelial-mesenchymal transition process.^36^ ZEB1 high expression is associated with tumor onset and progression, metastasis, and treatment resistance of various malignancies.^37^, ^38^, ^39^ As a transcription factor, ZEB1 can activate or repress the transcription of target genes by recruiting different cofactors.^40^ Interestingly, ZEB1 can activate PD-L1 transcription by binding to the promoter of PD-L1 and promote tumor immune escape.^41^ ZEB1 was increased in AML and ZEB1 was negatively mediated by miR-128–3p targeting. Importantly, ZEB1 overexpression could reduce the influence of circ-RERE downregulation on PD-L1 expression. In addition, it was found that up-regulation of ZEB1 could reverse the inhibitory effect of down-regulation of circ-RERE on the proliferation, autophagy and immune escape of AML cells and the promotion of apoptosis.

This study has several limitations. First, the clinical samples were obtained from a single center, which may introduce selection bias, requiring validation in larger multicenter cohorts. Second, miR-128–3p may regulate additional target genes in AML, necessitating transcriptomic analyses to elucidate its complete molecular network. Furthermore, in the tumor immune microenvironment, targeting PD-L1 alone may be insufficient to fully restore T-cell function due to compensatory regulation by other checkpoints such as CTLA-4. Therefore, developing combination therapies with PD-L1 inhibitors and other immune checkpoint blockers holds significant clinical relevance.

## Conclusion

Circ-RERE level is elevated in AML, and it can modify miR-128–3p to promote ZEB1 expression, thereby promoting autophagy and immune escape in AML cells. This study explores the underlying mechanism of circ-RERE function in AML cells, providing a potential target for AML therapy.

## Ethics approval and consent to participate

This study was approved by the ethical committees of Yinzhou People's Hospital (n° 201902CN16). Written informed consent was obtained from all patients.

## Funding

Not applicable.

## Data availability statement

Data is available from the corresponding author on request.

## Conflicts of interest

The authors declare no conflicts of interest.

## Appendix Supplementary materials

Image, application 1

## Footnotes

Supplementary material associated with this article can be found, in the online version, at doi:10.1016/j.clinsp.2025.100850.
