# Supplementary material for: Circ-RERE promotes autophagy and immune escape in acute myeloid leukemia involving the miR-128-3p/ZEB1/PD-L1 axis
Source: Clinics (Sao Paulo). 2026 Mar 20;81:100850. doi: 10.1016/j.clinsp.2025.100850 (PMC13019951; doi:10.1016/j.clinsp.2025.100850)

**CLINICS-D-23-00644_Supplementary Material**

**Supplementary Figure 1 ZEB1 binds to the PD-L1 promoter.** Predicted ZEB1 binding sites in the PD-L1 promoter region by JASPAR database analysis (A‒B); Validation of ZEB1-PD-L1 promoter binding by ChIP-qPCR assay (C). Data are expressed as mean ± standard deviation (n = 3). * p < 0.05, ** p < 0.01, *** p < 0.001, **** p < 0.0001.


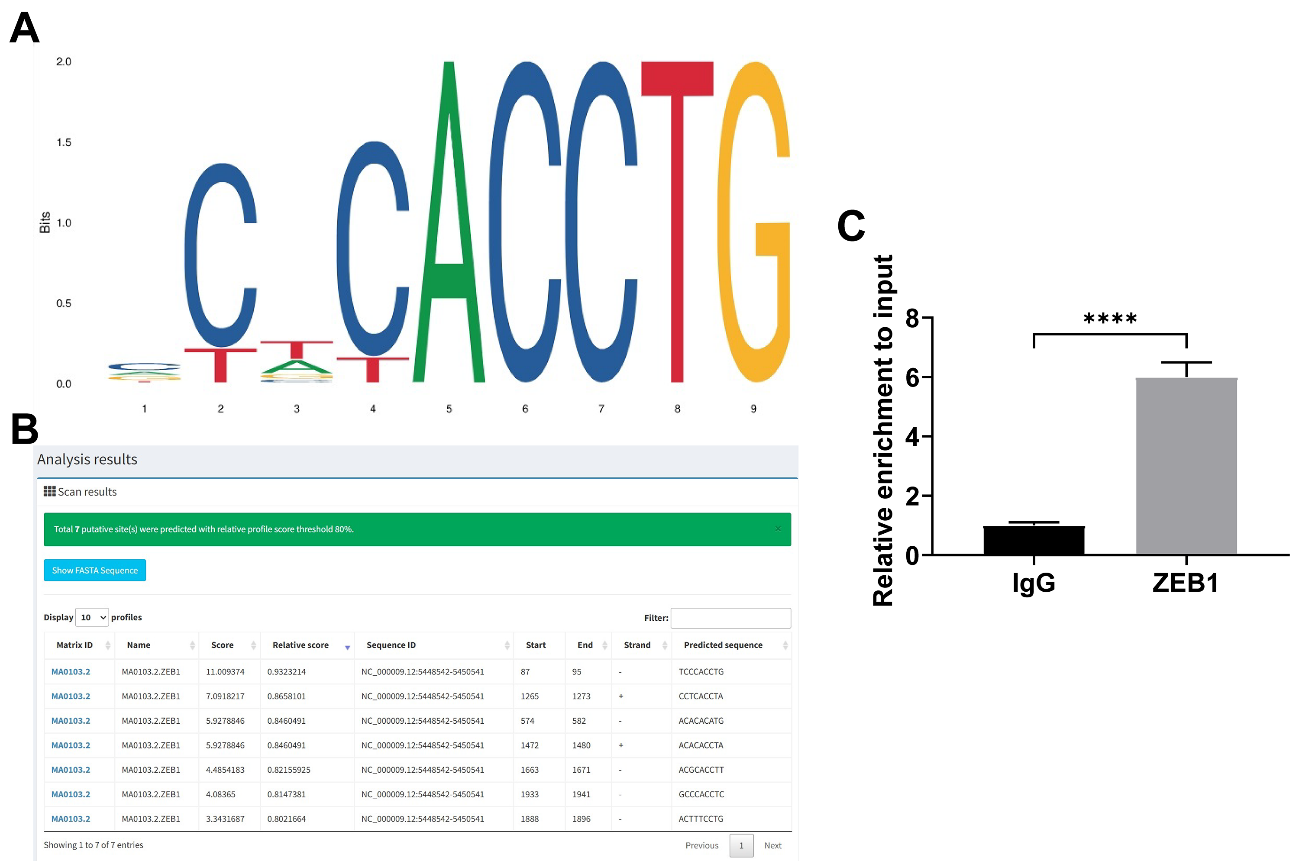

Supplement: Supplementary file 1 [file mmc1.docx]
